# Role of PI3K/AKT Signaling Pathway in Nucleus Pulposus Cells

**DOI:** 10.1155/2021/9941253

**Published:** 2021-07-01

**Authors:** Quan Xiao, Yun Teng, Changming Xu, Wei Pan, Hanshi Yang, Jiali Zhao, Quan Zhou

**Affiliations:** ^1^Department of Orthopaedic Surgery, The Affiliated Lianshui People's Hospital of Kangda College of Nanjing Medical Universty, Lianshui, Jiangsu, China; ^2^Department of Orthopaedic Surgery, The First Affiliated Hospital of Soochow University, Suzhou, Jiangsu, China; ^3^Department of Orthopaedic Surgery, The Affiliated Huai'an Hospital of Xuzhou Medical University, Huai'an, Jiangsu 223002, China

## Abstract

**Objective:**

To investigate the role of PI3K/AKT signaling pathway in nucleus pulposus (NP) cells.

**Methods:**

Nucleus pulposus (NP) cells were isolated from SD rat, and thereafter, passage three (P3) NP cells were divided into the following experimental groups: control, PI3K/AKT agonist IGF-1 (25 ng/ml, 50 ng/ml, and 100 ng/ml), and PI3K/AKT inhibitor LY294002 (5 *μ*M, 10 *μ*M, and 20 *μ*M). Flow cytometry and BrdU cell proliferation assays were performed to assess apoptosis and the proliferation rate of NP cells. Western blot analysis was performed to examine the protein expression level of Col II, Col X, Aggrecan, and MMP13.

**Results:**

PI3K/AKT inhibitor LY294002 increased the rate of apoptosis in NP cells when compared to the control and decreased the proliferation rate when compared to control. Moreover, LY294002 decreased the protein expression level of Col-II and Aggrecan in NP cells. At the same time, LY294002 increased the protein expression level of MMP13 and Col-X in NP cells. Through activating PI3K/AKT, IGF-1 increased the proliferation rate when compared to control and decreased the rate of apoptosis when compared to control. Additionally, IGF-1 decreased the protein expression level of MMP13 and Col-X and increased Col-II and Aggrecan in NP cells.

**Conclusion:**

The inhibition of PI3K/AKT signaling pathway accelerated the apoptosis of NP cells and facilitated the extracellular matrix degradation. However, the activation of PI3K/AKT pathway partly prevented the NP cell from apoptosis and promoted their proliferation. Meanwhile, its activation also delayed the loss of extracellular matrix.

## 1. Introduction

Intervertebral disc degeneration (IDD) is the main cause of lumbar disc herniation, spinal stenosis, and spondylolisthesis [1]. It severely affects public health and leads to a heavy economic burden on the society, which urges us to understand the pathogenic mechanism of IDD and find novel therapies [2]. The intervertebral disc structure is characterized by the annulus fibrosus around and nucleus pulposus (NP) located in the inner disc [3]. NP cells as the main components of the nucleus pulposus are responsible for the production and maintenance of extracellular matrix (ECM) containing collagen fibres and variety of proteoglycans especially aggrecan bearing compressive loads [4]. It is generally believed that IDD is partly caused due to the depletion of NP cells and degradation of ECM [5]. Reactive oxygen species resulting from Mitochondria dysfunction or inflammatory factors such as IL-1*β* and TNF-*α* tend to trigger apoptosis and disrupt the dynamic balance between ECM synthesis and degradation in NP cells [6, 7]. Apart from these, the abnormal activation or blockade of some signal pathways also participates in the process of apoptosis [8]. Therefore, targeting these pathways may be a strategy to inhibit apoptosis in NP cells to prevent and reverse IDD.

PI3K/AKT signal pathway involved in many biological processes involving cell survival, apoptosis, and inflammation [9]. PI3K has four molecular types including types I, II, III, and IV. Of those four types, PI3K type I is the most important and researched, which are heterodimers made up of a regulatory subunit (p85) and a catalytic subunit (p110) and participated in various bioactivities [10]. Downstream effectors AKT can be activated by phosphatidylinositol-3,4,5-trisphosphate (PIP3) which is formed by the transformation of phosphatidylinositol-4,5-bisphosphate (PIP2) [11]. Activated AKT can launch protein kinases, cell cycle regulators, and other downstream substrates of which mTORC1 plays a critical role in autophagy and can start autophagic signals to protect cells [12]. Most studies have shown that the PI3K/AKT signaling pathway is involved in the inflammatory process of NP cells [13]. In the inflammatory environment, activating PI3K/AKT pathway can protect NP cells from apoptosis and promote their proliferation [14, 15]. Also, the antioxidant capacity of NP cells also can be enhanced via AKT/autophagy [16]. However, recent studies attached their importance to the NP cells which have already degenerated. In these degenerated NP cells, the activation of PI3K/AKT is beneficial to NP cells. However, we did not know its specific role in normal NP cells and whether its activation is conductive to cell growth. Therefore, in this study, we aimed to investigate the role of the PI3K/AKT pathway in normal NP cells, thereby providing novel ideas for developing a treatment for IDD.

## 2. Materials and Methods

### 2.1. Isolation and Culture of Rat Nucleus Pulposus Cells

SD rats weighing 200-250 g were executed through the dislocation of cervical vertebra, socked in 75% ethanol for 5 min, and transferred onto the clean bench. NP tissues of the caudal intervertebral disc were harvested, cut into small pieces, washed in PBS with 10x penicillin-streptomycin twice, digested with 0.2% type II collagenase for 2 hours at 37°C, shook the tube every 20 minutes to promote the digestion, and filtered using a 100 *μ*m filter. After centrifugation at 1000 rpm for 5 min, the pellets were resuspended in an F12 medium containing 10% FBS and cultured in an incubator. The medium was changed for the first time after 3 days and, then, after every 2-3 days. The NP cells were passaged when cell confluency reached 80%~90%. Passage three (P3) NP cells were used for further experiments. All the animal experiments performed at the Affiliated Huai'an Hospital of Xuzhou Medical University were approved by the animal experimentation committee.

### 2.2. Cell Culture and Treatment

F12 medium mixed with 10% (*v*/*v*) fetal bovine serum, 100 U/ml penicillin-streptomycin were used to culture NPCs at 37°C and 5% CO_2_. For Western blotting and flow cytometry assays, the NPCs in advance were seeded into 10 cm dish and treated with IGF-1 (25 ng/ml, 50 ng/ml, and 100 ng/ml, PeproTech, American) or LY294002 (5 *μ*M, 10 *μ*M, and 20 *μ*M, MedChemExpress, American) when the confluency of cells reached 70%.

### 2.3. Detection of Apoptosis Using Flow Cytometry

After culturing P3 NP cells under different conditions for 48 h, the cells were digested with 0.2% trypsin, washed thrice with 1 × PBS, and incubated with Annexin V-FITC and PI (5 *μ*l) in the dark for 15-20 min at room temperature. Apoptosis in NP cells was detected through flow cytometry analysis within 1 h.

### 2.4. Detection of Cell Proliferation Using Flow Cytometry

P3 NP cells were seeded into the 96-well plate and cultured in a CO_2_ incubator for 60 h, followed by the addition of Brdu reagent to a final concentration of 50 *μ*M. After culturing for another 12 h, the cells were harvested and washed with 1 × PBS. After discarding the supernatant, the cells were suspended in 50 *μ*l staining buffer and, then, incubated with Brdu reagent for 20 min at room temperature in the dark. Thereafter, the cells were washed with 1 ml staining buffer and centrifuged at 1500 rpm for 5 min. After discarding the supernatant, 100 *μ*l of fixed permeable reagent was added to the tubes that were incubated for 15-30 min in the dark at room temperature, following which 1 ml of elution buffer was added and centrifuged at 1500 rpm for 5 min. The supernatant was discarded, and the cells were incubated with 100 *μ*l of DNase (300 *μ*g/ml) at 37°C for 1 h. Then, the cells were washed with 1 ml of elution buffer, centrifuged at 1500 rpm for 5 min, and incubated with 1.3 *μ*l of anti-Brdu antibody for 20 min at room temperature in the dark. Finally, the cells were washed with 1 ml of elution buffer and centrifuged at 1500 rpm for 5 min. The cells were suspended in 300 pL of elution buffer for detection using flow cytometry.

### 2.5. Western Blot Analysis

P3 NP cells cultured in IGF-1 or Rapamycin after 24 hours were washed by cold PBS three times and then lysed using the RIPA buffer (Beyotime, China). The solution was collected in the tube and centrifuged at 14000 g, 4°C for 5 minutes. The supernatant was kept and moved to the new tube. The protein concentration was determined by BCA Kit (ThermoFisher Scientific, American). The 5x loading buffer added to the supernatant was boiled. About 30 *μ*g of samples per well were subjected to SDS-PAGE and transferred onto the polyvinylidene fluoride membranes. The membranes were blocked in blocking buffer (Beyotime, China) and incubated with primary antibodies against AKT (1 : 1000, CST, American), p-AKT (1 : 1000, CST, American), Collagen II (1 : 2000, abcam, American), Collagen X (1 : 200, abcam, American), and *β*-actin (1 : 2000, Beyotime, China) overnight at 4°C. The membranes were washed for 5 minutes three times after the incubation of primary antibodies. The membranes were incubated with Horseradish peroxidase-conjugated goat anti-rabbit antibodies (1 : 10000, abcam, American) used as secondary antibodies for 1 hour at room temperature and washed for 30 minutes three times. Protein bands were detected using the ECL reagent kit (ThermoFisher Scientific, American), the signal intensities were quantified using the Image J software, and the results were normalized to *β*-actin and compared to the control group. All experiments were repeated three times.

### 2.6. Statistical Analysis

Data were expressed as mean ± standard deviation and analyzed using SPSS20.0. One-way analysis of variance (ANOVA) was used to assess the comparison between the groups, followed by LSD test. *P* < 0.05 was considered as statistically significant.

## 3. Results

### 3.1. PI3K/AKT Inhibitor LY294002 Slows down Cell Growth in NP Cells

**Figure 1:**
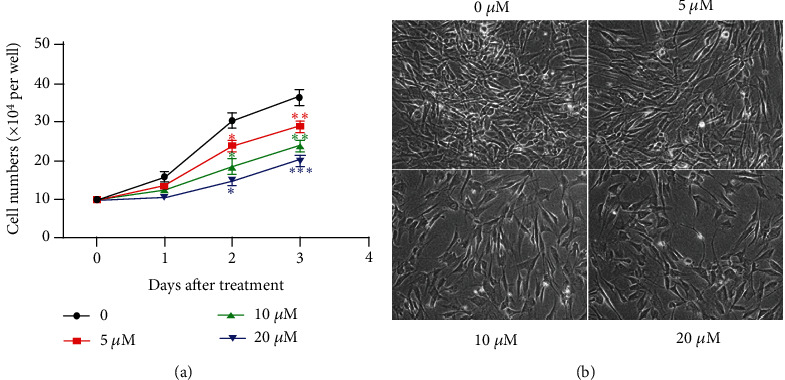
PI3K/AKT inhibitor LY294002 inhibits NP cell growth. (a) Changes in NP cell number in response to LY294002 at different doses. (b) Morphological changes in NP cells in response to LY294002 at different doses under the same scale. (^∗^*p* < 0.05, ^∗∗^*p* < 0.01, ^∗∗∗^*p* < 0.001, ^∗∗∗∗^*p* < 0.0001).

P3 NP cells were incubated with PI3K/AKT inhibitor LY294002 at different doses for 72 h. Cell number was assessed under a microscope at three different time points, 24 h,48 h, and 72 h. Data revealed that PI3K/AKT inhibitor LY294002 significantly decreased the number of NP cells at any time, especially after 48 h. Meanwhile, under the action of LY294002, the number of NPCs reduces in a dose-dependent manner and 20 *μ*M of LY294002 exerted the strongest effect on NPCs proliferation, which indicated that blocking PI3K/AKT is detrimental to NPCs growth (Figure 1(a)). Micromanaging NPCs also proved our view due to a dense population of cells in normal conditions and scarce cells with 20 *μ*M of LY294002 under the same scale (Figure 1(b)).

### 3.2. LY294002 Promotes Apoptosis and Inhibits Proliferation in NPCs

**Figure 2:**
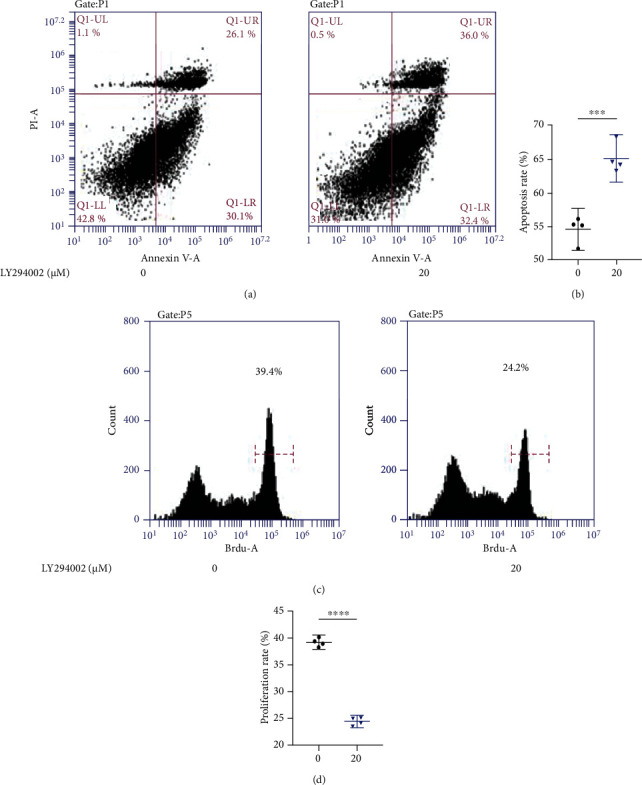
PI3K/AKT inhibitor LY294002 promotes apoptosis and decreases proliferation of NP cells. (a, b) Changes in the rate of NP cells apoptosis in response to LY294002. (c, d) Changes in the rate of proliferation of NP cells in response to LY294002. (^∗^*p* < 0.05, ^∗∗^*p* < 0.01, ^∗∗∗^*p* < 0.001, ^∗∗∗∗^*p* < 0.0001).

Flow cytometry analysis was performed to analyze apoptosis in NP cells treated with PI3K/AKT inhibitor LY294002 whose concentration is 20 *μ*M. Compared to the control group, LY294002 increased the rate of NP cell apoptosis from 56.12% to 68.4% (Figures 2(a) and 2(b)). BrdU assay was performed to detect the proliferation of NP cells in response to LY294002. Compared to the control group, LY294002 decreased the rate of NP cell proliferation from 39.4% to 24.2% (Figures 2(c) and 2(d)).

### 3.3. PI3K/AKT Inhibitor LY294002 Changes the NP Cells Protein Expression

**Figure 3:**
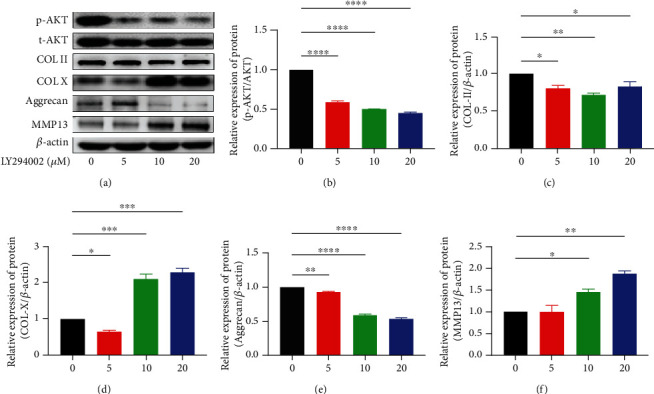
(a–f) Western blot showing that PI3K/AKT inhibitor LY294002 downregulated the ratio of p-AKT to t-AKT and the protein expression of Col-II and aggrecan as well as upregulated the protein expression of MMP13 and Col-X in NP cells. Protein expression was normalized to *β*-actin. (^∗^*p* < 0.05, ^∗∗^*p* < 0.01, ^∗∗∗^*p* < 0.001, ^∗∗∗∗^*p* < 0.0001).

Western blot experiments were performed to detect the protein expression of NPCs. The signal intensity of total AKT (t-AKT) and phosphorylated AKT (p-AKT) was quantified to calculate the ratio. In the presence of LY294002, p-AKT/t-AKT was significantly reduced in a dose-dependent manner. The protein expression of Col-II and aggrecan which are the hallmark of the extracellular matrix synthesis are significantly decreased in NP cells in response to PI3K/AKT inhibitor LY294002 and the protein expression of ECM enzyme MMP13 increased. LY294002 also led to a rise in the expression of Col-X which is highly expressed in degenerated NP tissue. (Figures 3(a)–3(f)).

### 3.4. PI3K/AKT Agonist IGF-1 Promoted Proliferation and Inhibited Apoptosis in NP Cells

**Figure 4:**
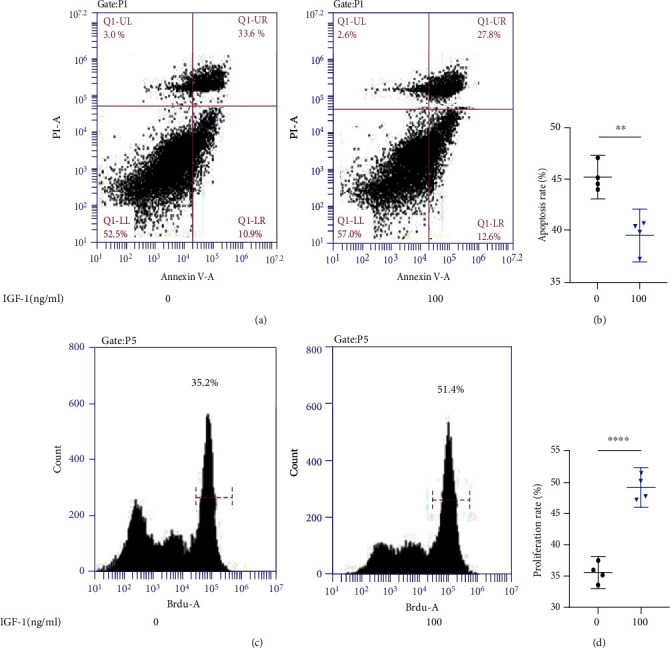
PI3K/AKT agonist IGF-1 promotes NP cell proliferation and inhibits apoptosis. (a, b) Changes in the rate of NPCs apoptosis in response to IGF-1. (c, d) Changes in the rate of proliferation of NP cells in response to IGF-1. (^∗^*p* < 0.05, ^∗∗^*p* < 0.01, ^∗∗∗^*p* < 0.001, ^∗∗∗∗^*p* < 0.0001).

Flow cytometry analysis was performed to analyze apoptosis in NP cells treated with 100 ng/ml of IGF-1. Compared to the control group, IGF-1 decreased the rate of apoptosis in NP cells from 44.5% to 40.4% (Figures 4(a) and 4(b)). BrdU assay was performed to detect the proliferation of NP cells in response to AKT agonist IGF-1. Compared to the control group, IGF-1 increased the rate of NP cell proliferation from 35.2% to 51.4% (Figures 4(c) and 4(d)).

### 3.5. IGF-1 Affects the Protein Expression in NPCs

**Figure 5:**
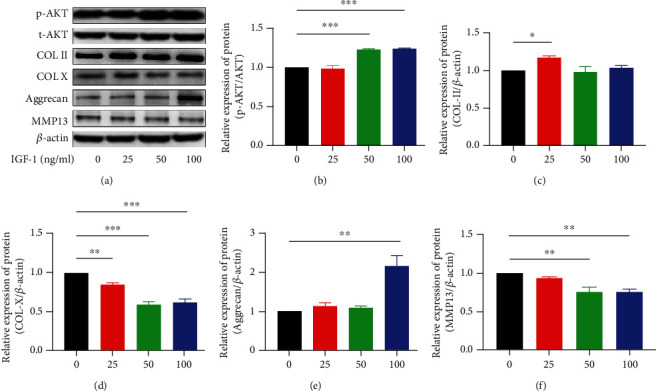
(a–f) Western blotting showed that PI3K/AKT agonist IGF-1 upregulated the ratio of p-AKT to t-AKT and the protein expression of Col-II and aggrecan as well as downregulated the protein expression of MMP13 and Col-X in NP cells. Protein expression was normalized to *β*-actin. (^∗^*p* < 0.05, ^∗∗^*p* < 0.01, ^∗∗∗^*p* < 0.001, ^∗∗∗∗^*p* < 0.0001).

Western blot experiments confirmed that the ratio of p-AKT and t-AKT significantly increased under the treatment of IGF-1 over 50 ng/ml. The protein expression level of Col-II was upregulated obviously under 25 ng/ml of IGF-1, but other concentrations did not show obvious influence on Col-II protein expression. Beyond this, 100 ng/ml IGF-1 significantly increases aggrecan protein expression. However, IGF-1 downregulated the expression of MMP13 and Col-X protein in a dose-dependent manner (Figures 5(a)–5(f)).

## 4. Discussion

A large number of studies have shown that the PI3K/AKT signaling pathway plays an important role in various cellular activities, including cell survival, cell cycle, inflammation, and apoptosis [17]. Therefore, we speculated that the PI3K/AKT signaling pathway might be involved in the degeneration of NP cells which in turn result in IDD.

Both exogenous and endogenous factors cause apoptosis in NPCs which mostly are regulated by PI3K/AKT signaling pathway [8]. For now, a mass of studies has identified the aberrant transcription of genes which may activate or block PI3K/AKT pathway are related to the degenerated NPCs in IDD patients. Sirt1, as a deacetylase depending on NAD^+^, whose mRNA and protein level decreased markedly in degenerated NP tissue and cells, inhibits the activation of caspase3 and apoptosis, yet when NPCs were transfected with Sirt1 siRNA or cocultured with LY294002, the action of sirt1was restrained. This remained us PI3K/AKT pathway may be an essential ritual in sirt1 protecting NPCs [18]. On the contrary, Yu et al. found USP15 expression was elevated in degenerative NPCs. USP15 interacting with miR-338-3P via blocking PI3K/AKT pathway enhance the protein expression related to apoptosis such as bax in NPCs and this function can be reversed by IGF-1 [19]. All these studies prompt us to value PI3K/AKT pathway. We utilized LY294002 and IGF-1 generally recognized as PI3K/AKT inhibitor and agonist to detect PI3K/AKT pathway how to realize function in normal NPCs. We observed that LY294002 significantly influences cell growth according to cell counting and microscopic observation, and this effect also showed a dose-dependent manner. The 20 *μ*M of LY294002 inhibitory is the strongest, and the number of NPCs is dramatically reduced. Next, we use flow cytometry to test the apoptosis and proliferation of NPCs in the presence of LY294002 and IGF-1. The results revealed that LY294002 promoted NPCs apoptosis and repressed their proliferation which probably give rise to NPCs degeneration. Conversely, PI3K/AKT agonist IGF-1 was found to protect NPCs from apoptosis and promote proliferation in NP cells. Thus, keeping PI3K/AKT pathway activation to a certain extent is helpful for NPCs growth and survival, which is line with Harris Pratsinis et al. found that simulating NPCs by IGF-1 and other two growth factors could encourage cell proliferation [20]. Also, Liu et al. identified that the levels of IGF-1 in LDD patients' serum remarkably decreased compared with normal population [21]. This hints that the state of the PI3K/AKT pathway also altered in degenerative NPCs which interfered with NPCs survival condition.

To verify the activation or blocking of the P13K/AKT pathway, we performed Western blot analysis to assess the expression of total AKT (t-AKT) and phosphorylated AKT (p-AKT). The consequence showed that LY294002 and IGF-1 at different doses did not affect t-AKT protein expression levels whereas LY294002 gradually decreased the p-AKT and p-AKT/AKT ratio in a dose-dependent manner. On the contrary, IGF-1 increased the p-AKT and p-AKT/AKT ratio in a dose-dependent manner. These data indicate that LY294002 inhibited, and IGF-1 activated P13K/AKT signaling pathway in NPCs.

On the basis of the detection of phosphorylated AKT, we assayed the protein expression related to ECM synthesis and degradation involving Col-II, Col-X, aggrecan, and MMP13. Col-II and Col-X are critical components of the intervertebral disc [22]. Studies have shown that the level of Col-II decreases in human IDD [23, 24]. Col-X is a short-chain collagen that is transiently expressed by the calcified cartilage and intervertebral disc tissue during growth and development [25]; however, it disappears from the intervertebral disc tissue after birth and is detectable during the incidence of IDD. In a previous study, the expression pattern of Col-X in intervertebral discs across different ages has been evaluated [26], and the researchers detected the expression of Col-X in both IDD and advanced disc herniation. Therefore, it can be stated that Col-II is negative, while Col-X is positive, correlated with the degree of degeneration in NP cells [27]. In the present study, the 25 ng/ml of IGF-1 treatment significantly increased the expression of Col-II in NP cells, and other concentrations did not make an obvious difference to Col-II expression. Moreover, LY294002 decreased Col-II nearly in a dose-dependent manner. Consistently, IGF-1 significantly decreased Col-X and LY294002 increased Col-X. Tian et al. showed that in anoxic and nutritionally deprived environments, the Col-II gene expression significantly reduced which IGF-1 almost helped return to the normal and LY294002 worsened the situation in NP mesenchymal stem cells [28]. Our study confirmed that in normal conditions, NPCs also lose Col-II and obtain Col-X due to antagonizing P13K/AKT pathway. Proteoglycan, as the major structure of the ECM interacting with water, gives disc tissue the ability to withstand compression [29]. The loss of proteoglycan especially aggrecan tends to appear in early disc degeneration [30]. Our western blot experiment analysis showed IGF-1 in 100 ng/ml obviously increased aggrecan protein expression and LY294002 in a dose-dependent manner downregulated aggrecan expression. Cheng et al. proved P13K/AKT modulates aggrecan gene expression through SOX9 and p300 from a gene perspective [31]. Our findings confirmed aggrecan protein expression also can be regulated by P13K/AKT. In degenerative response, ECM degradation cannot be separated from matrix metalloproteinase such as MMP1, MMP3, and MMP13 [7]. So we choose MMP13 to evaluate degrading enzyme activity, and the results were identified that LY294002 upregulated MMP13 expression and IGF-1 downregulated MMP13 expression which consistent with the findings of Lu et al. that in the osteoarthritis rat model via activating P13K/AKT, MMP13 secretion was suppressed [32]. What is more, Liu et al. also argued MMP3 can be decreased in the treatment of IGF-1 [21].

P13K/AKT signal pathway may play a protective role in NP cells. We speculated that its inhibition contributing to accelerating the degradation of NP cell matrix and stunting NP cell growth is related to the induction of early IDD. The disturbance of P13K/AKT due to the abnormal expression of miRNA including miR-138-5p and miR-27 participate in the process of IDD which has been demonstrated in a handle of studies [33, 34]. By contrast, maintaining the activation of P13K/AKT perhaps benefits to delaying IDD by protecting NPCs against apoptosis and ECM degradation not only in inflammatory or oxidative environment but in normal conditions.

However, our study has some limitations. First, we treated NP cells with IGF-1 and LY294002, which not only affect PI3K/AKT but other signal pathways that we cannot get rid of may be disturbed. Secondly, we only conducted single cell experiment and did not make animal experiments. So, more studies should be required to explore the function of PI3K/AKT in the degeneration of normal NP cells.

Collectively, the PI3K/AKT signaling pathway plays a critical role during the degeneration of NP cells. Manipulation of the PI3K/AKT pathway could promote, alleviate, and even inhibit NP cell degeneration. These findings provide a novel therapeutic strategy and target for developing the treatment for IDD.

## Data Availability

The data used to support the findings of this study are available from the corresponding author upon request.
